# Impaired Liver Size and Compromised Neurobehavioral Activity are Elicited by Chitosan Nanoparticles in the Zebrafish Embryo Model

**DOI:** 10.3390/nano9010122

**Published:** 2019-01-19

**Authors:** Haissam Abou-Saleh, Nadin Younes, Kashif Rasool, Manaf H. Younis, Rafael M. Prieto, Hadi M. Yassine, Khaled A. Mahmoud, Gianfranco Pintus, Gheyath K. Nasrallah

**Affiliations:** 1 Department of Biological and Environmental Sciences, College of Arts and Sciences, Qatar University, Doha 2713, Qatar; hasaleh@qu.edu.qa; 2 Department of Biomedical Science, College of Health Sciences, Qatar University, Doha 2713, Qatar; ny1204022@student.qu.edu.qa; 3 Biomedical Research Center, Qatar University, Doha 2713, Qatar; hyassine@qu.edu.qa; 4 Qatar Environment and Energy Research Institute (QEERI), Hamad Bin Khalifa University (HBKU), Qatar Foundation, Doha 2713, Qatar; krasool@hbku.edu.qa (K.R.); kmahmoud@hbku.edu.qa (K.A.M.); 5 Department of Public Health, Qatar University, Doha 2713, Qatar; my1512859@student.qu.edu.qa; 6 ZeClinics SL, PRBB (Barcelona Biomedical Research Park), 08003 Barcelona, Spain; zeminyana@gmail.com; 7 Department of Biomedical Sciences, University of Sassari, 07100 Sassari, Italy

**Keywords:** zebrafish, chitosan, nanoparticles, organs specific toxicity

## Abstract

The use of chitosan nanoparticles (ChNPs) in various biological and environmental applications is attracting great interest. However, potential side effects related to ChNP toxicity remain the major limitation hampering their wide application. For the first time, we investigate the potential organ-specific (cardiac, hepatic, and neuromuscular) toxicity of ChNPs (size 100–150 nm) using the zebrafish embryo model. Our data highlight the absence of both acute and teratogenic toxic effects of ChNPs (~100% survival rate) even at the higher concentration employed (200 mg/L). Although no single sign of cardiotoxicity was observed upon exposure to 200 mg/L of ChNPs, as judged by heartbeat rate, the corrected QT interval (QTc, which measures the time between the start of the Q wave and the end of the T wave in the heart's electrical cycle), maximum cardiac arrest, and ejection fraction assays, the same dosage elicited the impairment of both liver size (decreased liver size, but without steatosis and lipid yolk retention) and neurobehavioral activity (increased movement under different light conditions). Although the observed toxic effect failed to affect embryo survival, whether a prolonged ChNP treatment may induce other potentially harmful effects remains to be elucidated. By reporting new insights on their organ-specific toxicity, our results add novel and useful information into the available data concerning the in vivo effect of ChNPs.

## 1. Introduction 

Chitosan nanoparticles (ChNPs) have emerged as a pivotal instrument in many applicative fields, including chemistry, water treatment, aquatic herbicide, bioengineering, disease detection, and drug delivery [1,2,3,4,5]. Indeed, among their several intrinsic properties, ChNPs have shown to possess phytoremediation, antibacterial, and anti-pesticide effects [3,4,5,6,7,8], which make them a prominent tool in applied environmental science. In addition, due to their many advantages, including simple preparation, physicochemical stability, and improved bioavailability, these polymer-based nanomaterials have attracted ample attention in the biomedical theragnostic field [9,10]. In fact, because of their nanosized and chemical versatility, chemically engineered tissue-targeted ChNPs can readily cross cell membranes, conductive tissues (such as blood and lymphatic vessels), and the brain barrier [10]. In addition, ChNPs have been shown to exert antitumor activity, either by boosting the body’s immune system or by interfering with the tumor growth [11,12]. 

The wide range of ChNP applications urges the need to accurately investigate potential ChNP toxicity on both aquatic life and high vertebrate animals, including humans. Although the safe use of ChNPs is still an area of debate, many studies consider chitosan a safe polymer that can be employed in several in vivo applications, such as tissue engineering, drug delivery, enzyme immobilization, and wound dressing [2,13]. Indeed, low molecular weight (MW) chitosan can be directly cleared by the kidney in vivo, while high MW chitosan is first enzymatically degraded and then eventually cleared from the body as the low MW chitosan [12]. Nevertheless, some extent of toxicity has been reported in other studies, both in vitro and in vivo [14], thus emphasizing the necessity of further studies to unravel this specific aspect of Chitosan.

Zebrafish and their embryos are extensively used as tools to assess the toxicity of a compound, not only in the environmental context (aquatic in particular) but also to predict the toxic effects of compounds in humans [15,16]. This animal model has been widely used in toxicological research to assess the toxicity of novel drugs in vivo [12]. Because of the great similarities in cellular structure, signaling processes, anatomy, and physiology between zebrafish and other high-order vertebrates, particularly in the early stages of development [16]**,** this model can be useful in providing toxicological information translatable to humans [17]. Although some studies on ChNP toxicity in Zebrafish have been performed [18,19,20], to our knowledge, there are no reported data concerning potential organ-specific and long-term toxicity of ChNPs. Therefore, using zebrafish as a model system, this study aims to design and implement a number of organ-specific (heart, muscles, nervous system, and liver) toxicity assays to comprehensively evaluate any potential in vivo adverse effects of ChNPs. In this regard, we decided to investigate a range of concentrations (25–200 mg/L) consistent with the acute toxicity rating scale provided by the U.S. Fish and Wildlife Service (USFWS) [21].

## 2. Materials and Methods

### 2.1. Materials

All materials were purchased from Sigma Aldrich (St. Louis, MO, United States) unless indicated otherwise. Low MW (LMW) chitosan (50–190 KDa, based on viscosity) powder with 75%–85% degree of deacetylation (Cat No., 448869-250G) was used. Dimethyl sulfoxide (DMSO) was used as negative control (NC) in all assays; dimethylaminobenzaldehyde (DEAB), a competitive inhibitor of aldehyde dehydrogenases known to cause mortality and teratogenic phenotype in zebrafish embryos, was used as positive control (PC) in acute toxicity assays [22]. Haloperidol, an antipsychotic drug, was used as a PC in cardiotoxicity assays [22,23,24]. In addition, 1-Methyl-4-phenyl-1,2,3,6-tetrahydropyridine hydrochloride (MPTP), a neurotoxic drug that causes permanent symptoms of Parkinson's disease, was used as a PC in neurotoxicity assays [25]. Paracetamol (APAP) was used as a PC in hepatotoxicity assays [26,27]. A 60X stock solution of embryo media (E3 media) was prepared from 8.765 g NaCl, 380 mg KCl, 1.19 g MgSO_4_, and 1.765 g CaCl_2_ dissolved in 0.5 liters of MilliQ water. 

### 2.2. Chitosan Nanoparticle Preparation

ChNPs were synthesized by ionic crosslinking reaction at different initial chitosan concentrations of 0.05%, 0.1%, 0.25%, and 0.5 %. LMW chitosan was dissolved in an aqueous solution of 1% (*w*/*w*) acetic acid to form a 1.0 mg/mL chitosan solution and filtered (pore size 0.45 µm, Millipore, Burlington, MA, United States) to remove insoluble particles. Tripolyphosphate (TPP 0.5 mg/mL) was dissolved in ultrapure water and filtered. TPP solution was added dropwise to the chitosan solutions, having different initial chitosan concentrations and TPP ratios. The reaction was carried out for 10 min, and the resulting suspension was washed three times with deionized (DI) water to remove any remaining TPP. Finally, the obtained ChNPs pellet was resuspended into DI water for the needed applications.

### 2.3. Chitosan Nanoparticle Characterization

ChNPs were characterized by Zetasizer, X-ray diffraction (XRD), Fourier-transform infrared spectroscopy (FTIR), scanning electron microscopy (SEM), and UV-Vis spectroscopy. A Zeta phase analysis light-scattering (ZetaPALS) analyzer (Malvern Instruments, Malvern, United Kingdom) was used to measure the electrophoretic mobilities (EPMs) of the ChNPs. The zeta potentials were calculated from the average EPMs. The ChNPs sizes were measured by dynamic light scattering (DLS) using a ZetaPALS analyzer. To determine how the ionic strength impacted the nanoparticles size, we prepared nanoparticle suspensions containing different salt concentrations and immediately put them into disposable cuvettes. XRD was recorded using a Bruker D8 Advance (Bruker AXS, Billerica, MA, United States). SEM analyses were performed using an FEI Quanta 650 FEG scanning electron microscope after samples gold sputter coatings.

### 2.4. Zebrafish Embryo Culture

Wild-type and transgenic green fluorescent proteins (GFP) AB zebrafish strains (*Danio rerio*) were used. The fish were left in a 14 h light/10 h dark cycle with a water temperature of 28 °C. Fertilized eggs were rinsed and were collected in Petri dishes containing E3 egg medium. After 3–4 h, unfertilized and unhealthy embryos were discarded. At 24 h post-fertilization (hpf), the healthy fertilized embryos were dechorionated by adding 1.0 mg/mL of pronase. The embryos were incubated for 10 min and swirled, so the chorion becomes soft. Then the embryos were washed three times with fresh E3 media and moved to six-well plates containing a fresh E3 medium. Finally, the E3 medium was replaced by fresh E3 medium containing the different concentrations of drugs to be tested in different assays. In this study, we followed the national and international guidelines for the use of zebrafish embryos to carry out all the experiments in accordance with animal protocol guidelines required by the ZeClinics, Spain, and Qatar University and Policy on Zebrafish Research established by Department of Research in the Ministry of Public Health, Qatar.

### 2.5. Acute Toxicity Assays

The toxicology of ChNPs was investigated by acute toxicity assays adapted by the Organization of Economic Co-operation and Development (OECD) guidelines for testing chemical toxicity (N° 203 and 236). Healthy dechorionated embryos were transferred to a six-well plate containing the following treatments prepared in fresh E3 media: (i) four different concentrations (25, 50, 100, 200 mg/L) of ChNPs, (ii) the PC DEAB (0.1, 1, 10, 100, and 1.0 μM), and (iii) the NC 0.1% DMSO. Embryos were incubated at 28 °C from 24 hpf up to 96 hpf. Cumulative survival was recorded at three time point intervals (48, 72, and 96-hpf). In addition, all embryos were observed every day for teratogenic abnormalities, such as pigmentation, body deformities, heart edema, heartbeats, yolk edema, scoliosis, and movement. The median lethal dose (LC50) was calculated by fitting a sigmoidal curve to mortality data with a 95% confidence interval. A total of 100 embryos were used for each tested dose condition of the ChNPs, and 20 embryos for each DEAB and 0.1% DMSO concentrations.

### 2.6. Neurobehavioral Toxicity Evaluation

In order to determine the effect of the ChNPs on the embryos’ nervous systems, we assessed their swimming activity (distance traveled) in response to environmental stimuli, such as dark and light exposure [28]. Briefly, 24-hpf healthy embryos were allowed to develop normally until 96 hpf at 28 °C, which is the time when the proper swimming activity is reached [29]. At 96 hpf, embryos were transferred to a 96-well plate (one embryo per well) containing the following treatments: (i) the NC DMSO, (ii) the PC 100 µM MPTP, and (iii) 200 mg/L ChNPs. Next, the embryos were incubated with each treatment until 120 hpf, and the locomotion activity was recorded up to 144 hpf. The potential neurobehavioral toxicity of ChNPs was determined by locomotion assessment using the EthoVision XT 11.5 tracking software and the DanioVision device (Noldus, Wageningen, Netherlands), which allows the tracking of individual zebrafish embryos during alternating dark and light cycles. The 96-well plate was placed into the DanioVision chamber, and the embryos were left for 20 min with light for acclimation. Next, embryo movements were recorded during a 10-min alternate light/dark cycle for a total of 50 min. Neurotoxicity was evaluated by comparing the above parameters between the treated and control groups. Due to circadian rhythms, all locomotion assays were performed from 13:00 pm onwards to ensure the steady activity of embryos [30].

### 2.7. Cardiotoxicity Assays

The cardiotoxicity assays were performed using the transgenic cardiac myosin light chain 2 gene-GFP zebrafish (Tg[cmLc:GFP]) strain. This strain expresses the green fluorescence proteins (GFP) in the cardiac myocytes, thus allowing a good quality of cardiac imaging. Briefly, 24-hpf healthy embryos were allowed to develop normally until 96 hpf, which is the time where the heart is usually fully developed [31]. At 96 hpf, embryos were incubated for 4 h at 28 °C with the following treatments: (i) the NC DMSO, (ii) the PC 10 µM haloperidol, and (iii) 200 mg/L ChNPs. Treated embryos were then anesthetized by immersion in 0.7 µM Tricaine methanesulfonate/E3 solution. For imaging, every embryo was positioned under the microscope using an agarose-based mold, and the fluorescent heart was recorded for 60 s [15]. Videos were acquired by high-speed cameras and analyzed with the ZeCardio® software for the presence of any heart dysfunctions. In particular, the following cardiac parameters were assessed: heart rate; QTc corrected interval (Framingham formula: QTc = QT + 0.154 (1 − RR)) [32]; cardiac arrest and ejection fraction (Ef%) = ((DD − SD)/DD) × 100. DD); ventricle diastolic diameter (max dilatation); SD; and ventricle systolic diameter (max contraction)].

### 2.8. Hepatotoxicity Evaluation 

The hepatotoxicity assays were performed using the Tg[cmLc:GRP] transgenic AB strain of zebrafish. This strain expresses the RFP in the hepatocytes, thus allowing a good quality of liver imaging. To evaluate potential ChNP hepatotoxicity, the following parameters were assessed in the zebrafish liver: liver size, to measure necrosis and hepatomegaly, and yolk retention and steatosis, to measure liver lipid metabolism. At 96 hpf, embryos were incubated for an additional 32 h at 28 °C with the following treatment: (i) the NC DMSO; (ii) the PC 2% EtOH (for steatosis assessment), 2640 µM APAP, or 2% EtOH (yolk retention and necrosis assessment); and (iii) 200 mg/L ChNPs. 

#### 2.8.1. Liver Area Analysis

For the liver size measurement, embryos were fixed in 4% paraformaldehyde for 4 h at RT and then washed three times with PBS. Fixed embryos’ fluorescent livers were imaged with a fluorescence stereomicroscope (Olympus MVX10) using a digital camera (Olympus DP71). RFP filtered images were analyzed using cell^D and the FIJI software. 

#### 2.8.2. Detection of Steatosis and Yolk Retention 

Steatosis and yolk retention were evaluated by Oil Red O staining (Sigma-Aldrich, St. Louis, MO, United States). Zebrafish embryos were stained as previously described [33]. Briefly, the skin pigment of the fixed embryos was removed by incubation with 0.1 mL of 5% sodium hypochlorite bleaching solution for 20 min, followed by five washes with PBS at RT. Then, the bleached embryos were submerged in 85% propylene glycol (PG) (Sigma-Aldrich, United States) for 10 min, and then in 100% PG for another 10 min, before staining them with Oil Red 0.5% in 100% PG (overnight, at RT and with a gentle rocking). Oil Red O stained embryos were then washed in 100% PG for 30 min, 85% PG in PBS for 50 min, and finally 85% PG in PBS for 40 min. Next, the embryos were washed with 1x PBS before adding 80% glycerol. Finally, bright field images were taken to detect both steatosis and yolk retention. For the analysis of steatosis, embryos were considered to be positive when three or more round lipid droplets were visible within the hepatic parenchyma [22]. Then, the percentage of steatosis was calculated by dividing the number of embryos showing steatosis with the total number of embryos observed. For yolk retention, embryos showing a strong red signal in the yolk area were considered positive. The percentage was calculated by dividing the number of positive embryos with the total number of embryos.

### 2.9. Statistical Analysis

Results were expressed as average ± SEM (standard error of the mean). Statistical evaluation of differences between experimental group means was performed using one-way analysis of variance (ANOVA), followed by the Dunnet test. The Chi-square test was used for the hepatotoxicity assays (steatosis and yolk retention) to compare the significance between the percentages. Outliers were eliminated by using the Graph Pad software. Significance (*): *p* < 0.05; (**): *p* < 0.01. 

## 3. Results and Discussion 

### 3.1. Chitosan Nanoparticle Characterization

**Figure 1:**
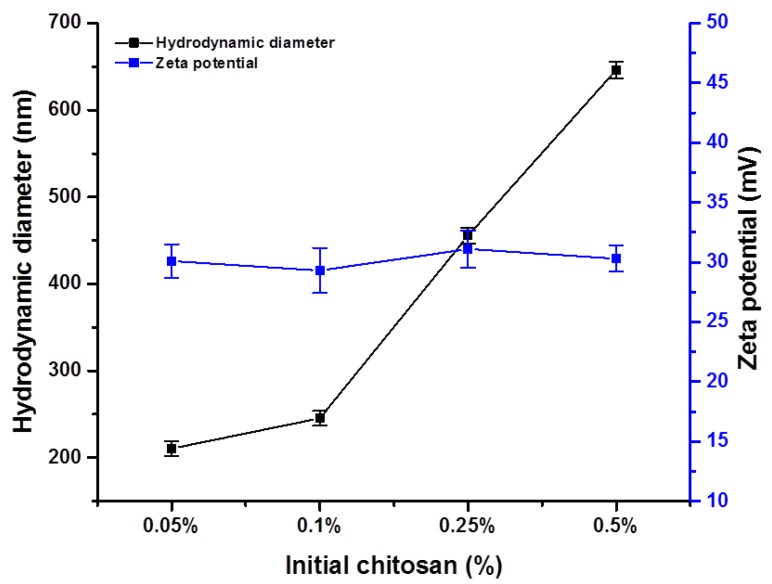
Hydrodynamic diameter and zeta potential of synthesized chitosan nanoparticles (ChNPs) at different initial chitosan concentrations.

Figure 1 depicts the hydrodynamic diameter and zeta potential of the synthesized ChNPs at different initial chitosan concentrations measured by dynamic light scattering. As the chitosan concentration was increased from 0.05% to 0.5%, the ChNPs hydrodynamic diameter increased from 120 nm to 646 nm. Below a certain concentration of chitosan (2.0 mg/mL as reported), the intermolecular hydrogen bonding attraction and the intermolecular electrostatic repulsion are in equilibrium. Therefore, in this concentration range, as chitosan concentration increases, chitosan molecules approach each other with a limit, leading to a limited increase in intermolecular cross-linking; thus, larger but still nanoscale particles are formed.

**Figure 2:**
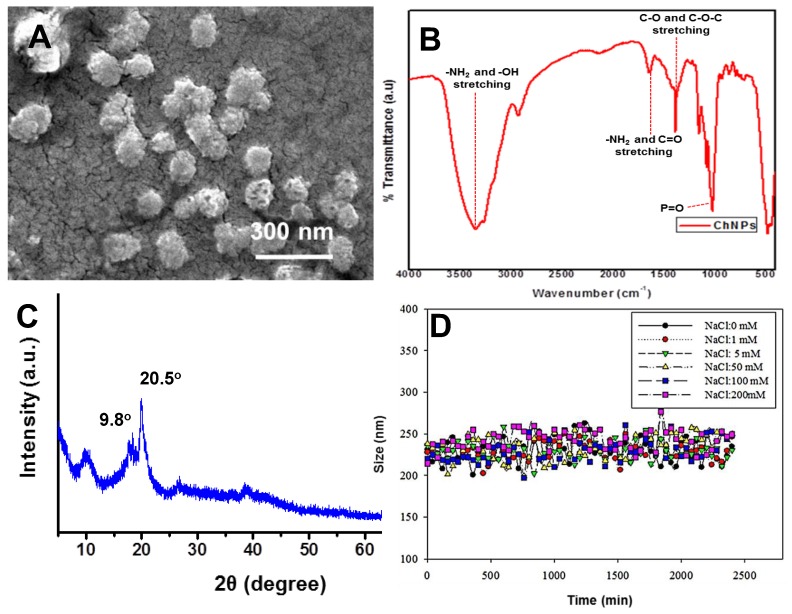
(**A**) SEM image, (**B**) FTIR spectra, and (**C**) X-ray diffraction patterns of ChNPs. (**D**) Impact of ionic strength on the stability of ChNPs, measured as hydrodynamic diameter by dynamic light scattering (DLS).

The primary size of selected ChNPs was in the range of 100–150 nm, as measured by SEM (Figure 2A). Figure 2B shows the FTIR spectra of the ChNPs. A characteristic band at 3435 cm^−1^ was attributed to the –NH_2_ and –OH groups stretching vibrations. The bands at 1640 cm^−1^, were attributed to the –NH_2_ bending vibration, whereas 1396 and 1078 cm^−1^ were assigned to CH_3_ symmetrical deformation, and the C–O stretching vibrations (C–O–C) of Ch, respectively. Figure 2C depicts the XRD spectra of ChNPs. The broad diffraction peaks at 2θ = ~9.80° and ~20.5° in the XRD pattern of ChNPs showed the semi-crystalline nature of the synthesized nanoparticles. Table 1 gives physicochemical characteristics of ChNPs, with the smallest size selected for further characterization and toxicity studies.

Zeta potential is an important tool for understanding the state of the nanoparticle surface and predicting the long-term stability of the nanoparticle. However, there was no significant effect of chitosan concentration found on the zeta potential, which was about 28 ± 2 for all chitosan compositions. The magnitude of the zeta potential is predictive of colloidal stability [34]. Nanoparticles with zeta potential values greater than +25 mV or less than −25 mV typically have high degrees of stability. Therefore, our results revealed the ChNPs as stable in colloidal suspension with a zeta potential of more than +25.0.

The physicochemical properties and colloidal stability of the nanoparticles probably largely determine their mobility, bioavailability, and toxicity to the living organisms. The effect of the salts’ concentration on the stability of the ChNPs was examined by varying the initial sodium chloride concentrations and maintaining the solution pH and ChNPs concentration at 7 ± 02 and 20 mg/L, respectively. It was found that synthesized nanomaterials were stable at a higher salt concentration and there was no impact on the nanomaterials’ hydrodynamic diameter (Figure 2D). 

### 3.2. Chitosan Nanoparticles Do Not Affect Zebrafish Embryos Survival

**Figure 3:**
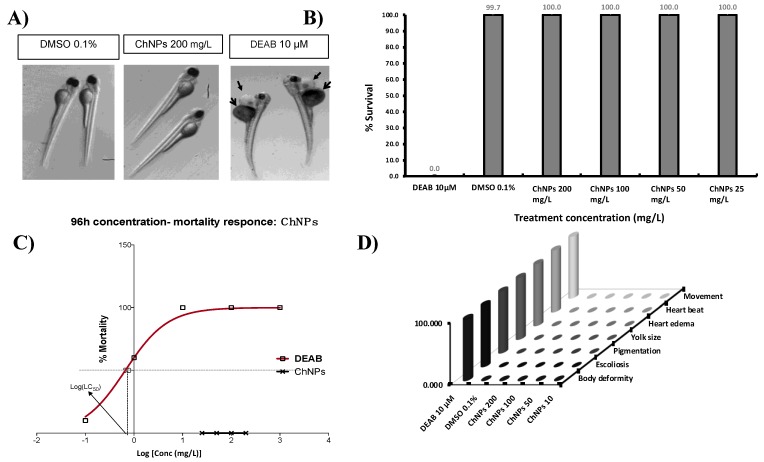
(**A**) Acute toxicity representative pictures obtained with a stereomicroscope at magnification X = 0.63. Note the deformed embryos in dimethylaminobenzaldehyde (DEAB): short size, scoliosis heart edema (closed arrow), and yolk edema (open arrow). (**B**) Cumulative survival/mortality. (**C**) Mortality response curve of different concentrations of DEAB and ChNPs. (**D**) Teratogenic phenotypes analysis. DEAB: Dimethylaminobenzaldehyde, positive control for toxicity and teratogenicity.

Embryos are more sensitive to external compounds and chemicals than larval or adult zebrafish [35]. Therefore, we chose the embryonic period from 24 to 96 hpf as the administration time to study potential ChNP toxicity. The percentage of cumulative survival was measured at 96 hpf, which is the recommended observation time [22]. According to Figure 3B, the no-observed-effect concentration (NOCE) (i.e., <20% mortality) for DEAB was 0.1 µM, as this concentration of DEAB showed a cumulative mortality of only 10% (2 out of 20 embryos were dead), and the rest of the embryos (90%) did not show any teratogenic effects (e.g., morphological or physiological abnormalities). While the low-observed-effect concentration (LOEC) (i.e., =>20% mortality) for DEAB was 1.0 µM, showing cumulative mortality of 60% (12 of 20 embryos were dead). At 10 µM of DEAB, the embryos showed severe teratogenic effects, such as deformities in the heart and yolk sac (Figure 3A), and were unable to survive up to 96 hpf. According to the sigmoidal curve (Figure 3C), the LC50 value for DEAB was calculated to be 0.665 µM (r^2^ ≈ 0.999). Concerning the ChNPs treatments, no mortality, morphological, or physiological abnormalities were observed (Figure 3D) at any dose and time employed, therefore NOEC, LOEC, and LC50 for ChNPs could not be calculated. These results suggest that the hypothetical LC50 for ChNPs would be much higher than 200 mg/L (Figure 3C). The current data demonstrated that ChNPs, at the least at concentrations as high as 200 mg/L, failed to affect zebrafish gross embryonic development. 

It is believed that ChNPs’ toxicity is related to particles size and concentration, chemical composition, and ambient conditions. Indeed, nanoparticles’ toxicity appears to increase with the decrease in their size [36]. To the best of our knowledge, only three articles studying the effect of ChNPs on zebrafish embryos have been published [18,19,20], and none of them performed a comprehensive investigation of potential organ-specific toxic effects. Indeed, Hu et al. tested the potential ChNP toxicity in relation to the particle sizes (200 and 340 nm), showing that the 200 nm ChNPs are more toxic toward zebrafish embryos compared to the 340 nm. The 200 nm ChNPs were able to cause 100% mortality and severe teratogenic phenotypes at a concentration as low as 40 mg/L, while the 340 nm ChNPs, although at less of an extent as compared to the 200 nm, were able anyway to promote significant mortality and teratogenic phenotypes as compared to the untreated embryos. Our current results are in disagreement with those obtained by Hu et al. In fact, although we employed smaller ChNPs sizes (100−150 nm) than those used by Hu et al., we were unable to record any toxic effects or teratogenic phenotypes at concentrations as high as 200 mg/L. On the other hand, our current results are consistent with those reported by Wang et al., indicating that 200 mg/L of ChNPs failed to cause significant embryos mortality (<10%), even when a smaller ChNP size (84.86 nm) was employed. Under our current experimental conditions, the LOEC for ChNPs could not be determined. Therefore, we used the highest tested concentration (200 mg/L) in the subsequent toxicity assays performed. 

### 3.3. Chitosan Nanoparticles Induce Neurobehavioral Impairment In Zebrafish Embryos 

**Figure 4:**
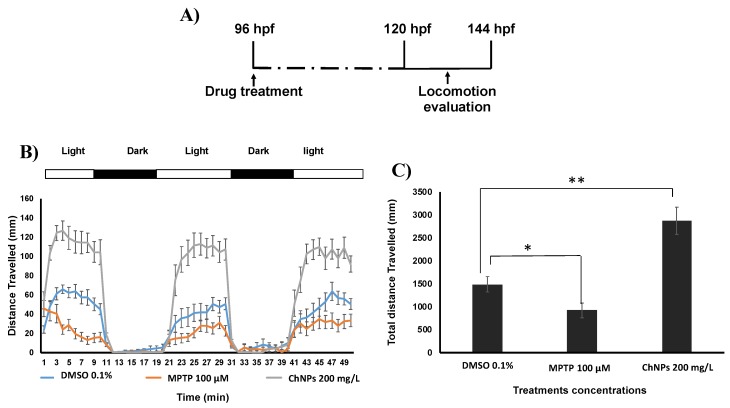
Locomotion assay. (**A**) Scheme of the experimental procedure. (**B**) Average of distance travelled (mm) per min measured by DanioVision. A group of 14 embryos was used for each treatment. (**C**) Average of cumulative distance travelled (mm) by the treated group after exposure to 50 min dark/light cycle. MPTP: 1-Methyl-4-phenyl-1,2,3,6-tetrahydropyridine hydrochloride, positive control for neurotoxicity.

Zebrafish are widely used as a model for studying neurological diseases and behavioral analysis [37]. To gain insight on ChNPs’ potential neurobehavioral toxicity, we investigated their effect of on the embryos’ nervous system development by quantifying the swimming activity of the embryo exposed to two illumination conditions. Consistently with a previous article [20], our data indicate that ChNPs-treated embryos display an abnormal increase in the locomotion behavior (hyperactivity) compared to the untreated ones (Figure 4B,C). However, the data reported by Yuan et al. [20] along with our current findings both show increased rather than a decreased behavior activity, suggesting that ChNPs can adversely affect the embryos’ nervous system, but is unlikely to affect their muscle development.

### 3.4. Chitosan Nanoparticles Do Not Affect Cardiac Functions

**Figure 5:**
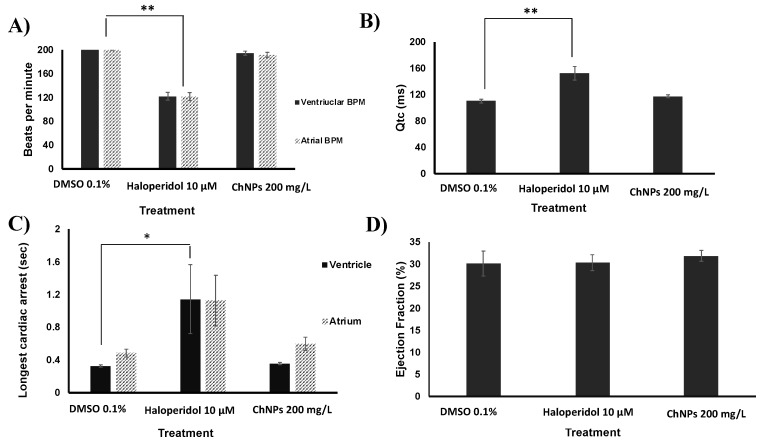
Cardiotoxicity assays. (**A**) Heartbeat rate. (**B**) QTc corrected interval. (**C**) Maximum cardiac arrest. (**D**) Ejection fraction. Ten embryos were used for each treatment concentration.

Among the advantages of zebrafish as an experimental model is the similarity of their cardiovascular system to that of mammals [38]. At 120 hpf, zebrafish embryos were exposed to 200 mg/L ChNPs and DMSO, and 10 µM haloperidol was used as positive control (PC) for cardiotoxicity. Four parameters were assessed for the cardiotoxicity evaluation: (i) heart rate in beat per min (BPM); (ii) the corrected QT interval (QTc), which measures the time between the start of the Q wave and the end of the T wave in the heart's electrical cycle; (iii) cardiac arrest; and (iv) ejection fraction. A compound such as the ChNPs, for instance, would be considered cardiotoxic if able to induce a significant difference from the negative control (0.1% DMSO) in one of the above-mentioned parameters. The positive control haloperidol was able to induce a significant decrease in the heart rate as compared to negative control DMSO (Figure 5A). This result is consistent with previous data showing that haloperidol is able to induce bradycardia in humans and zebrafish [24]. Similar to the ability of haloperidol to produce QT prolongation in humans [39], our current data indicates a significant prolongation of this cardiac parameter in ChNPs-treated zebrafish as compared to the untreated ones (Figure 5B). However, ChNPs failed to affect the normal heartbeat frequency of both the ventricle and the atrium, and were unable to elicit any impairment of the QTc when compared to untreated embryos. As expected, haloperidol-treated embryos showed significantly longer cardiac arrests as compared to DMSO (Figure 5C). Concerning ChNPs, the longest cardiac arrests at the dose of 200 mg/L were 0.32 and 0.4 s for the ventricle and the atrium, respectively, which were both not significant in comparison to DMSO. Regarding the ejection fraction (Figure 5D), no significant difference was detected between DMSO and 200 mg/L ChNPs. In summary, ChNPs were unable to induce any cardiotoxicity or developmental delay even at high concentrations or after long exposure time (four days).

### 3.5. Chitosan Nanoparticles Induce Liver Size Impairment in Zebrafish Embryos

**Figure 6:**
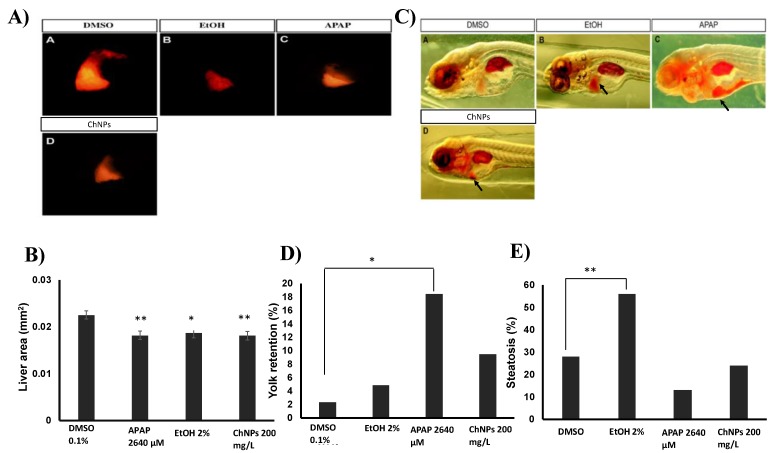
(**A**) Representative images for the RFP liver area after each treatment. (**B**) Quantification and measurement of the RFP liver area (mm^2^) for the controls and all the tested compounds. (**C**) Representative images for steatosis and yolk retention. Arrow (↗) in EtOH treatment indicate liver steatosis (absorbed ORO stain by the liver), and arrows (↗) in paracetamol (APAP) and ChNP treatments indicate increased yolk retention (absorbed ORO stain by the yolk). (**D**) Percentages of embryos showed yolk retention and **(E**) steatosis. Thirty embryos were used for each treatment in every experiment. *, significantly different from DMSO (*p* < 0.05); **, significantly different from DMSO (*p* < 0.01).

Due to the close similarity of mammals' molecular and cellular processes, Zebrafish embryos have been recently used in liver research, including hepatotoxicology [26,27,40,41]. Since most nanoparticles accumulate in the liver [42], hepatotoxicity tests are essential to assess potential ChNP toxicity. After 120 hpf, the embryos’ liver is fully functional and able to metabolize external nutrients from the environment [43]. The yolk sac of the zebrafish embryos consists of 70% lipids, which are mainly metabolized in the liver [44]. Thus, excess lipid accumulation in the yolk can be used as an indirect indication of impaired liver function. If the liver function is impaired, the yolk lipid metabolism and absorption will be delayed, which in turn will result in lipid retention in the yolk [45]. Hepatotoxicity is primarily caused by metabolic process dysfunction, which requires a certain period of time to occur. Therefore, we performed our experiments 32 h after embryos’ treatment with ChNPs (at 128-hpf). Here, we performed three different hepatotoxicity assays. First, we assessed changes in the liver size in response to different treatments (Figure 6A,B). Similar to the hepatotoxicity positive control ethanol, 200 μg/mL ChNPs-treated embryos showed a significant decrease in both liver size and shape as compared to DMSO. Further, embryo exposure to a different positive control (APAP) [46] also displayed severe changes in liver size compared to DMSO, indicating severe liver necrosis. Second, we also assessed the percentage of embryos that developed abnormal ORO yolk retention in response to the treatments. No significant difference in ORO yolk retention was found between the DMSO and the ChNP-treated embryos (Figure 6D). However, consistent with a previous study [22], APAP-treated embryos showed a significant increase in ORO yolk retention when compared to DMSO. 

Steatosis directly reflects an impairment of the normal synthesis and elimination of triglycerides within the hepatocytes [47]. Thus, the effect of ChNPs on hepatocyte lipid metabolism was performed by a steatosis assay, which measures the amount of lipid retention in the liver in response to treatments. The ChNP-treated embryos (Figure 6E) did not show significant signs of steatosis compared to the untreated ones. However, 56% of the ethanol-treated embryos showed signs of steatosis, a result consistent with data presented by Passeri et al. [48]. 

## 4. Conclusions

Our study for the first time presents data that comprehensively investigates the organ-specific toxicity of ChNPs in vivo in the zebrafish embryo model. The performed toxicity test indicated that ChNPs were unable to induce teratogenic phenotypes and embryo death at the used concentrations. Moreover, ChNP-treated embryos displayed normal heart physiology, thus indicating the absence of cardiotoxic effects. Nevertheless, ChNPs exerted remarkable neurotoxic effects and significantly impaired the liver size, suggesting these nanoparticles are potentially harmful to both the brain and liver physiology of zebrafish embryos. We believe that our results add new insights into the available data concerning the in vivo effect of ChNPs and provide useful information concerning the use of ChNPs in polymer-based nanomaterials technology. Whether ChNPs may affect other organs at higher concentrations or after prolonged exposition needs to be determined, and will be the object of future study.

## Figures and Tables

**Table 1:** Physicochemical characteristics of ChNPs.

Properties	Technique	Unit	Value
Primary size	TEM	nm	100–150
Particle size in DI water	DLS	nm	210 ± 6
Phase and structure	XRD	-	Semi-crystalline
Shape/morphology	TEM	-	Spherical, hexagonal
Surface area	BET	m^2^/g	11.7
pH_PZC_	DLS	-	28 ± 2.6
